# Mutational fitness landscape of human influenza H3N2 neuraminidase

**DOI:** 10.1016/j.celrep.2022.111951

**Published:** 2023-01-05

**Authors:** Ruipeng Lei, Andrea Hernandez Garcia, Timothy J.C. Tan, Qi Wen Teo, Yiquan Wang, Xiwen Zhang, Shitong Luo, Satish K. Nair, Jian Peng, Nicholas C. Wu

**Affiliations:** 1 Department of Biochemistry, University of Illinois at Urbana-Champaign, Urbana, IL 61801, USA; 2 Center for Biophysics and Quantitative Biology, University of Illinois at Urbana-Champaign, Urbana, IL 61801, USA; 3 Carl R. Woese Institute for Genomic Biology, University of Illinois at Urbana-Champaign, Urbana, IL 61801, USA; 4 HeliXon Limited, Beijing 100084, China; 5 Department of Computer Science, University of Illinois at Urbana-Champaign, Urbana, IL 61801, USA; 6 Carle Illinois College of Medicine, University of Illinois at Urbana-Champaign, Urbana, IL 61801, USA; 7 Lead contact

## Abstract

Influenza neuraminidase (NA) has received increasing attention as an effective vaccine target. However, its mutational tolerance is not well characterized. Here, the fitness effects of >6,000 mutations in human H3N2 NA are probed using deep mutational scanning. Our result shows that while its antigenic regions have high mutational tolerance, there are solvent-exposed regions with low mutational tolerance. We also find that protein stability is a major determinant of NA mutational fitness. The deep mutational scanning result correlates well with mutational fitness inferred from natural sequences using a protein language model, substantiating the relevance of our findings to the natural evolution of circulating strains. Additional analysis further suggests that human H3N2 NA is far from running out of mutations despite already evolving for >50 years. Overall, this study advances our understanding of the evolutionary potential of NA and the underlying biophysical constraints, which in turn provide insights into NA-based vaccine design.

## In brief

Lei et al. measure the replication fitness effects of >6,000 single amino acid mutations in human influenza H3N2 neuraminidase, which is emerging as a vaccine target for broadened protection. The results provide insights into the evolutionary potential and constraints of influenza neuraminidase.

## Graphical Abstract

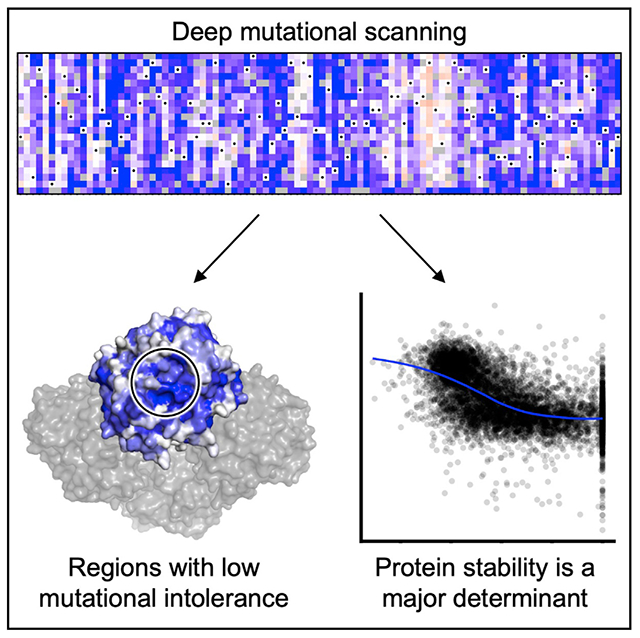

## INTRODUCTION

Despite the practice of social distancing and masking during the COVID-19 pandemic, influenza A virus continues to circulate in the human population. In fact, influenza H3N2 virus has been circulating in human for more than half a century without any sign of disappearing. Influenza A virus has two surface glycoprotein antigens, hemagglutinin (HA) and neuraminidase (NA). Compared with HA, which has traditionally been the focus of influenza vaccine development, NA is poorly characterized. Nevertheless, NA is emerging as an effective vaccine target due to studies that demonstrate anti-NA immunity as an independent correlate of protection^1–4^ as well as the recent discovery of broadly protective NA antibodies.^5^

NA is a homotetrameric type II transmembrane protein with an N-terminal cytoplasmic tail, a transmembrane domain, a hypervariable stalk region, and a C-terminal head domain. The head domain, which accounts for ~80% of NA protein sequence, contains an enzymatic active site that cleaves sialic acid to facilitate virus release and prevent virion aggregation.^6^ The enzymatic active site of influenza NA is also a target of several FDA-approved antivirals.^7^ Similar to HA, NA of human influenza A virus also undergoes antigenic drift.^8,9^ The major antigenic regions of NA are at the rim of its enzymatic active site.^10,11^ Our previous study on seven antigenic residues has demonstrated that charge balancing constrains the antigenic evolution of NA.^12^ More recently, we have also shown that epistasis between certain natural mutations of human H3N2 NA can be explained by protein stability.^13^ While these studies have helped understand the biophysical constraints of NA evolution, the mutational fitness landscape of NA remains largely elusive.

The development of deep mutational scanning, which combines saturation mutagenesis and next-generation sequencing, enables many mutations to be studied in a massively parallel manner and thus allows the mutational fitness landscape to be empirically determined.^14^ Recent deep mutational scanning studies of severe acute respiratory syndrome coronavirus 2 (SARS-CoV-2) receptor-binding domain have provided critical insights into the evolution and antibody escape of SARS-CoV-2^15–17^ as well as COVID-19 vaccine design.^18^ Similarly, deep mutational scanning has also been successfully applied to probe the mutational fitness landscape and antibody escape of influenza HA.^19–24^ However, most, if not all, deep mutational scanning studies of viral proteins were performed *in vitro* (i.e., non-animal systems). The correlation between *in vitro* fitness measurement and *in vivo* replication or transmission fitness is not well characterized but is fundamental to virus research.

In this study, we used deep mutational scanning to measure the fitness effects of >6,000 single amino acid mutations in the head domain of influenza H3N2 NA. The result enabled us to identify solvent-exposed regions that have low mutational tolerance, which are critical to vaccine development. Our analysis also demonstrated that protein stability is a major determinant of the mutational fitness landscape of NA. In addition, fitness measurements in our deep mutational scanning experiment, which was performed *in vitro*, correlated well with mutational fitness that was inferred from the natural sequences of human H3N2 NA (Spearman’s rank correlation = 0.59). This observation further allowed us to draw conclusion about the evolutionary potential of NA in circulating human H3N2 virus.

## RESULTS

### Deep mutational scanning of human H3N2 NA

We aimed to analyze the fitness effects of mutations in the NA of H3N2 A/Moscow/10/1999 (Mos99) using deep mutational scanning (Figure S1). Briefly, saturation mutagenesis was applied to generate all possible single amino acid mutations from residues 82 to 465 in Mos99 NA, which covered 99% of the head domain (residues 82 to 469). Of note, N2 numbering is used unless otherwise stated. The virus mutant library was then rescued by the influenza reverse-genetics system^25^ and passaged once in MDCK-SIAT1 cells, which could minimize the emergence of cell-adaptive mutation in NA.^26^ Using next-generation sequencing, the frequencies of individual mutations in the plasmid mutant library and the post-passaged mutant library were measured. Subsequently, the fitness of each mutation was computed as the normalized log_10_ enrichment ratio such that the mean fitness value of silent mutations was 0, whereas beneficial and deleterious mutations would have positive and negative fitness values, respectively.

**Figure 1.**
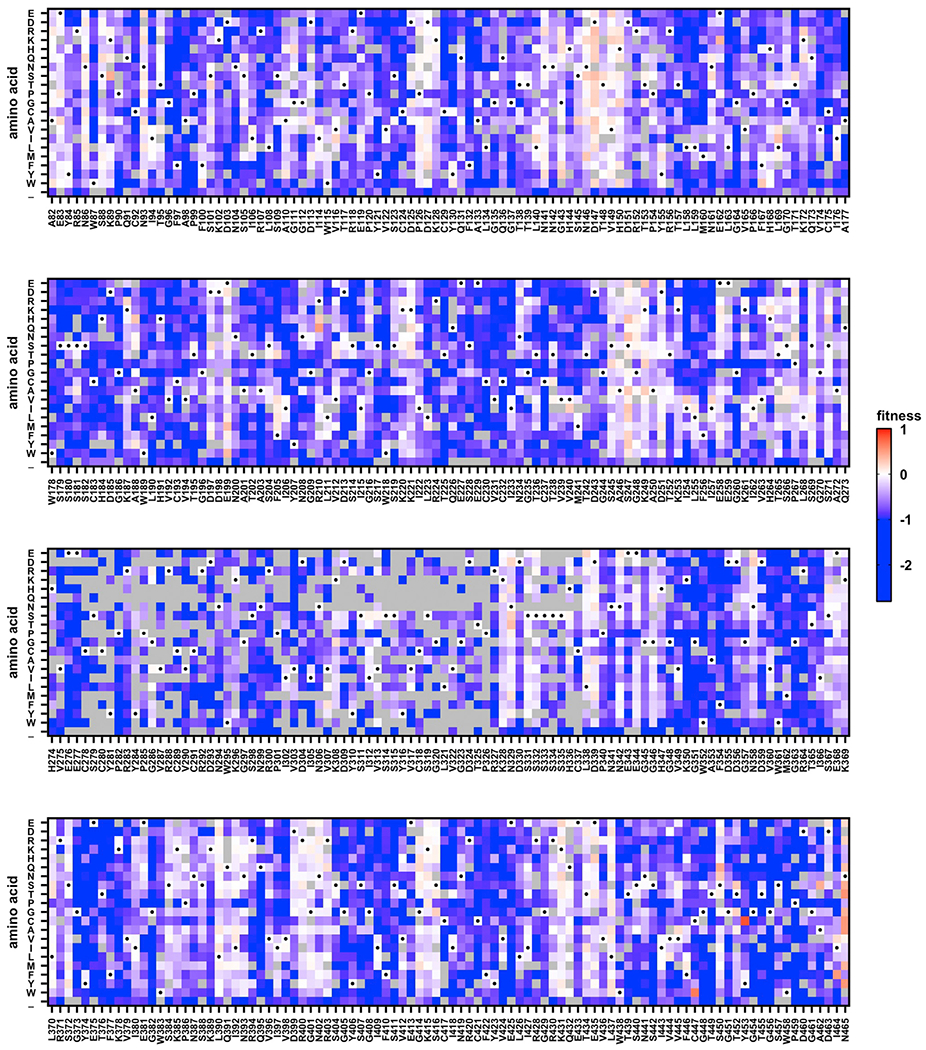
Deep mutational scanning of Mos99 NA head domain The fitness of individual mutations in the Mos99 NA head domain was measured by deep mutational scanning and is shown as a heatmap. Wild-type (WT) amino acids are indicated by a black circle. Mutations in gray were excluded in our data analysis due to low input count or high occurrence in the WT sample (see STAR Methods). See also Figures S1 and S2 and Table S1.

Our deep mutational scanning measured the fitness effects of 6,353 (87%) out of 7,296 all possible amino acid mutations across the 384 residues of interest (Figure 1; Table S1). A Pearson correlation of 0.84 was obtained between two biological replicates (Figure S2A), which was on the high end among deep mutational experiments using the recombinant influenza virus.^20,21,27,28^ The deep mutational scanning result here also correlated well with our previous study that focused on a seven-residue antigenic region in Mos99 NA^12^ (Figure S2B). Besides, the fitness distributions of silent and nonsense mutations had minimal overlap, further validating that fitness selection has taken place (Figure S2C).

### Biophysical determinants of mutational tolerance

We aimed to identify the biophysical constraints that determine the mutational tolerance of the NA head domain. Here, we defined the mutational tolerance at each residue as the average fitness of mutations at the given residue. Residues in the active site and antigenic regions were defined as previously described.^6,10^ Using the crystal structure of Mos99 NA (PDB: 7U4F) that was determined in our recent study,^13^ the remaining residues were categorized according to relative solvent accessibility (RSA). Residues in the protein core, the protomer-protomer interface, and surface were categorized as “buried,” “interface,” and “exposed,” respectively (see STAR Methods).

**Figure 2.**
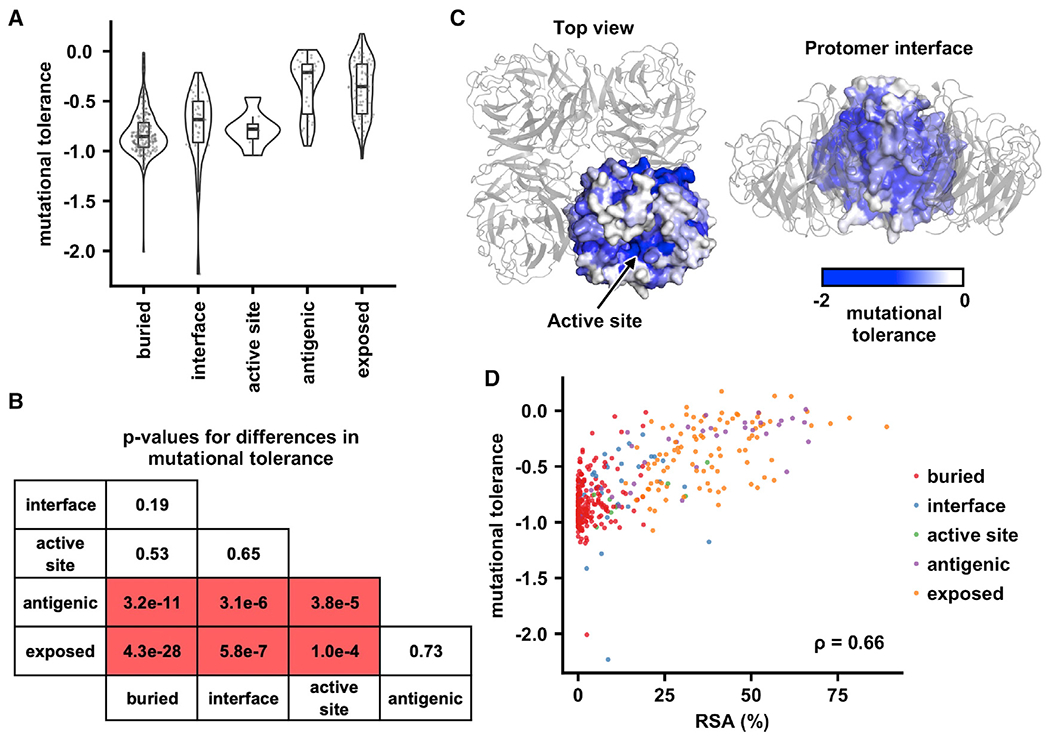
Biophysical determinants of mutational tolerance (A) Mutational tolerance was computed by averaging the fitness of mutations at each residue. The distribution of mutational tolerance for each residue category is shown as a violin plot and a boxplot. For the boxplot, the middle horizontal line represents the median. The lower and upper hinges represent the first and third quartiles, respectively. The upper whisker extends to the highest data point within a 1.5× inter-quartile range (IQR) of the third quartile, whereas the lower whisker extends to the lowest data point within a 1.5× IQR of the first quartile. Each data point represents the mutational tolerance of one residue. (B) The differences in mutational tolerance between residue categories were computed by two-tailed Student’s t tests. The p value for each pairwise comparison is shown. (C) Mutational tolerance of each residue is shown on Mos99 NA (PDB: 7U4F).^13^ One protomer is in surface representation with individual residues colored according to their mutational tolerance. The other three protomers are in gray cartoon representation. (D) The relationship between relative solvent accessibility (RSA) of each residue on the Mos99 NA tetramer (PDB: 7U4F)^13^ and its mutational tolerance is shown. Each data point represents one residue and colored according to the residue category. The Spearman’s rank correlation coefficient (ρ) is indicated. See also Figure S3 and Table S2.

Mutational tolerance of the exposed residues and antigenic regions was significantly higher than that of buried residues, interface residues, and active site residues (p ≤ 0.0001) (Figures 2A–2C). In contrast, mutational tolerance of the buried residues, interface residues, and active site residues did not differ significantly (p ≥ 0.19). Similarly, mutational tolerance of the exposed residues and antigenic regions did not differ significantly (p = 0.73). The mutational tolerance of different residue categories appeared to associate with their RSA. For example, residue categories with higher mutational tolerance, namely the exposed residues and antigenic regions, have higher RSA (Figure S3A). In fact, mutational tolerance correlated well with RSA for the 384 residues that were examined in our deep mutational scanning experiment (Spearman’s rank correlation = 0.66) (Figure 2D), consistent with similar studies on other proteins.^15,19^ Since mutations at residues with lower RSA are typically more destabilizing,^29–31^ our results indicate that folding and tetramerization stabilities are the major determinants of mutational tolerance in the NA head domain.

To further examine if the active site constrained the mutational tolerance of the surrounding residues, we determined the crystal structure of Mos99 NA in complex with sialic acid at 1.6 Å resolution (Table S2). Although the sialidase enzymatic activity is the major function of NA, mutational tolerance and the distance from active site only showed weak correlation (Spearman’s rank correlation = 0.18) (Figure S3B). The correlation did not improve if we only included those residues within 15 Å from the active site (Spearman’s rank correlation = 0.03) (Figure S3D). These observations substantiate the notion that optimal enzymatic activity of NA relies on the global conformational stability of the tetramer.^6,32,33^

### Clusters of surface residues with low mutational tolerance

**Figure 3.**
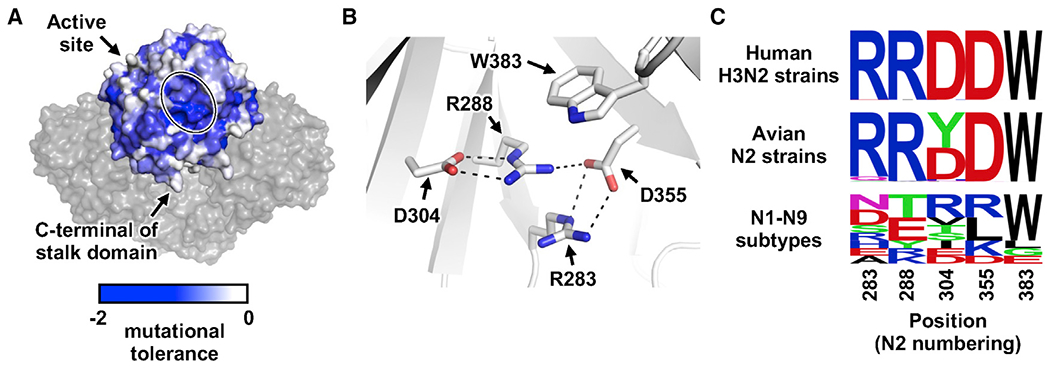
A cluster of residues with low mutational tolerance at the membrane proximal side of the NA head domain (A) Mutational tolerance of each residue is shown on Mos99 NA (PDB: 7U4F).^13^ One protomer is in surface representation with individual residues colored according to their mutational tolerance. The other three protomers are in gray surface representation. The C-terminal of the stalk domain, which connects the NA head domain to the membrane, is indicated. A cluster of residues with low mutational tolerance is circled. (B) Residues within the cluster in (A) are shown. Electrostatic interactions are represented by dashed lines. (C) The natural variants of NA residues 283, 288, 304, 355, and 383 (N2 numbering) are represented by sequence logos. Top: 58,937 human H3N2 strains from 1968 to 2020. Middle: 6,900 avian N2 strains. Bottom: representative strains from N1-N9 subtypes. See also Table S2.

When we projected the mutational tolerance data on the structure, two clusters of solvent-exposed residues with low mutational tolerance were identified (Figures 2C and 3A). The first cluster was the active site (see above; Figure 2C), which was expected due to its functional importance. The second cluster was at the membrane proximal side of the NA head domain (Figure 3A), consisting of five residues with low mutational tolerance, namely R283, R288, D304, D355, and W383 (Figure 3B). Together, these five residues have a solvent exposure surface area of 222 Å^2^ and form many interactions among themselves. Specifically, R283, R288, D304, and D355 form a network of electrostatic interactions. Furthermore, R288 forms π-stacking interactions with R283 and W383. As a result, the low mutational tolerance of these five residues is likely due to their critical role in NA folding stability. Consistently, these five residues are highly conserved in human H3N2 NA, albeit not across NA subtypes (Figure 3C).

### Correlation between protein stability and mutational fitness effects

**Figure 4.**
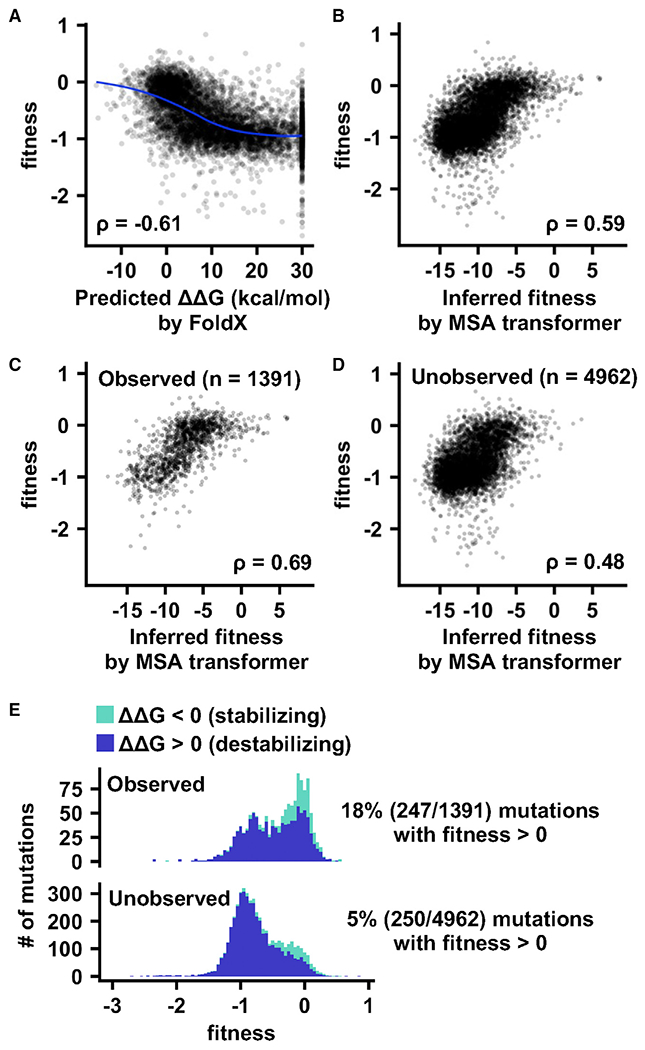
Deep mutational scanning result correlates with mutational stability and natural evolution (A) The relationship between predicted stability effect (ΔΔG) by FoldX^34,35^ and fitness measurement by deep mutational scanning is shown. Mutations with predicted ΔΔG >30 kcal mol^−1^ are shown as 30 kcal mol^−1^. A smooth curve was fitted by loess. (B) The relationship between fitness inferred from natural sequences using MSA transformer^40^ and fitness measured by deep mutational scanning is shown. The Spearman’s rank correlation coefficient (ρ) is indicated. (C and D) Same as (B) except only those mutations that are (C) observed in nature or (D) not observed in nature are shown. (E) Distributions of fitness effects of mutations that have been observed in nature (observed) and those that have not been observed in nature (unobserved) are shown. See also Figure S4 and Tables S2 and S3.

To systematically investigate how protein stability of NA influences the mutational fitness effects, we used FoldX^34,35^ to predict the protein stability effects of the 6,353 amino acid mutations that were examined in our deep mutational scanning experiment (Table S3). We observed a non-linear relationship between predicted mutational stability (ΔΔG) of the NA tetramer and mutational fitness (Figure 4A). Mutations that were predicted to be more stabilizing (ΔΔG < 0) tended to have a higher fitness. As the predicted stability decreased (i.e., ΔΔG increased), mutational fitness decreased and reached a plateau at a fitness of around −1, which was similar to the average fitness of nonsense mutations (Figure S2C). This observation indicates that once the NA protein is sufficiently destabilized, the virus would not be able to replicate. The correlation between predicted ΔΔG and mutational fitness (Spearman’s rank correlation = −0.61) confirms that protein stability is a major determinant of NA mutational fitness effects. Similar observations were made when a single NA protomer (i.e., monomeric form) was used for computing the predicted mutational stability, despite a slightly lower correlation with mutational fitness (Spearman’s rank correlation = −0.58) (Figure S3C). Overall, these results substantiate the notion that protein stability can help predict the functional effects of mutations^36^ and represents a major constraint of molecular evolution.^37–39^

### Correlation between natural evolution and mutational fitness effects

Next, we would like to examine if our deep mutational scanning result reflects the mutational fitness in the natural evolution of human H3N2 NA. Recently, the development of MSA transformer, which is a deep-learning-based protein language model, has allowed unsupervised inference of mutational fitness for any protein using multiple sequence alignment.^40–42^ Here, we applied MSA transformer to infer the fitness of mutations in the Mos99 NA using 66,562 NA sequences from human H3N2 strains that were isolated between 1968 and 2020. The inferred mutational fitness would therefore represent the mutational fitness in the natural evolution of human H3N2 NA (Table S3). For the 6,353 mutations that were examined in our deep mutational scanning experiment, the inferred fitness correlated well with the fitness measurement by deep mutational scanning (Spearman’s rank correlation = 0.59) (Figure 4B). The correlation was higher if only naturally observed mutations were considered (n = 1,391, Spearman’s rank correlation = 0.69) (Figure 4C). A moderate correlation could still be obtained if we only considered those mutations that were not observed in nature (n = 4,962, Spearman’s rank correlation = 0.48) (Figure 4D). A handful of naturally observed mutations had a high inferred fitness but were neutral in our deep mutation scanning experiment (horizontal tail in the top right of Figure 4C), potentially due to the positive selection pressures that were absent *in vitro*, such as herd immunity and transmission efficiency. Overall, despite the differences in selection pressures that are seen *in vitro* and in human population, our deep mutational scanning result largely aligns with the mutational fitness of human H3N2 NA in the natural setting.

### Unexplored functional sequence space in human H3N2 NA

As mentioned above, among the 6,353 mutations that were examined in our deep mutational scanning experiment, only 1,390 (22%) have been observed in naturally circulating human H3N2 NA. We aimed to assess if any of the remaining 4,963 (78%) mutations have the potential to emerge in circulating human H3N2 virus. According to our deep mutational scanning result, while the majority of these 4,963 mutations were deleterious to the virus, 251 had a fitness >0 (Figure 4E). Of note, mutations with fitness <0 have also been observed in nature (Figures 4C and 4E), although most of the highly deleterious ones (fitness < −1) are with low occurrence frequency (Figure S4). Given that our deep mutational scanning result correlates well with the mutational fitness in the natural setting (see above; Figure 4B), these observations suggest that circulating human H3N2 NA is far from running out of mutations.

## DISCUSSION

While NA is an emerging target for influenza vaccine development, the biophysical constraints on its evolution have not been entirely clear. In this study, we applied deep mutational scanning to study the mutational fitness landscape of influenza H3N2 NA. One important finding is that the *in vitro* fitness measurement in our deep mutational scanning experiment correlates well with the *in vivo* mutational fitness that is inferred from natural sequences. This observation has important implications for how to interpret *in vitro* fitness data of influenza NA mutants. However, we acknowledge that future studies need to examine if this correlation can be observed in other viral proteins.

HA and NA in human influenza virus undergoes antigenic drift independently.^8,9^ Previous deep mutational scanning studies have shown that the antigenic regions of HA have high mutational tolerance.^19,21^ Similarly, our work here demonstrates that the antigenic regions of NA also have high mutational tolerance. This result indicates that a broadly protective NA-based vaccine should focus the antibody response against immunosubdominant regions that have lower mutational tolerance. In fact, two clusters of solvent-accessible residues with low mutational tolerance are observed in this study. The first cluster is the active site, which is known to be highly conserved across influenza A and B^6^ and is a target of broadly protective antibodies.^5^ The second cluster, which is at the membrane-proximal side of the NA head domain, is highly conserved within the N2 subtype but not across different subtypes. Antibodies to this region, if any, should be at least somewhat cross-protective against antigenically distinct N2 strains and have a higher genetic barrier to escape compared with those that target the classic antigenic regions of NA. As the antigenicity of NA remains poorly understood, future studies should explore whether this second cluster of residues are targeted by human antibodies.

Recently, Moderna has initiated a phase 1/2 clinical trial of influenza mRNA vaccines that contain NA as a component (ClinicalTrials.gov: NCT05333289). The increasing importance of NA in public health urges continued efforts to characterize the evolutionary biology and biophysical properties of influenza NA, which will in turn facilitate the optimization of NA-based immunogens. The conformational stability of an immunogen often represents a key to achieve high vaccine effectiveness.^43^ Using deep mutational scanning data to identify stabilizing mutations has been demonstrated in a study on the SARS-CoV-2 receptor-binding domain.^18^ Since studies have shown that the conformational stability of NA can be improved by stabilizing mutations,^44,45^ we anticipate that our deep mutational scanning dataset will be instrumental for NA-based immunogen design.

### Limitations of the study

In this study, the Mos99 NA mutant library was rescued using the six internal segments from A/WSN/33 (H1N1) and HA from A/Hong Kong/1/1968 (H3N2). While the internal segments are unlikely to influence the mutational fitness landscape of NA, it is known that HA and NA functionally interact.^46–48^ Consistently, our recent study has shown that the mutational fitness landscape of HA can be influenced by NA.^49^ As a result, we acknowledge that our deep mutational scanning experiment may be slightly different if both HA and NA from Mos99 are used.

## STAR★METHODS

### RESOURCE AVAILABILITY

#### Lead contact

Information and requests for resources should be directed to and will be fulfilled by the lead contact, Nicholas C. Wu (nicwu@illinois.edu).

#### Materials availability

All plasmids generated in this study are available from the lead contact without restriction.

#### Data and code availability

- The read counts and fitness measurements from the deep mutational scanning experiment are in Table S1. Raw sequencing data have been submitted to the NIH Short Read Archive under BioProject: PRJNA857746. The X-ray coordinates and structure factors have been deposited in the RCSB Protein DataBank under accession code PDB: 8DWB.
- Custom python scripts for analyzing the deep mutational scanning data have been deposited to https://doi.org/10.5281/zenodo.7416749.
- Any additional information required to reanalyze the data reported in this paper is available from the lead contact upon request.

### EXPERIMENTAL MODELS AND SUBJECT DETAILS

#### Cell cultures

HEK 293T cells (human embryonic kidney cells, female) were maintained in DMEM medium (Thermo Fisher Scientific) supplemented with 10% fetal bovine serum (FBS, Thermo Fisher Scientific), 1x MEM non-essential amino acids (Thermo Fisher Scientific), and 100 U mL^−1^ of Penicillin-Streptomycin (Thermo Fisher Scientific). MDCK-SIAT1 cells (Madin-Darby canine hidney cells with stable expression of human 2,6-sialtransferase, female, Sigma-Aldrich) were maintained in DMEM medium supplemented with 10% FBS, 1x MEM non-essential amino acids, and 100 U mL^−1^ of Penicillin-Streptomycin. Sf9 cells (Spodoptera frugiperda ovarian cells, female, ATCC) were maintained in Sf-900 II SFM medium (Thermo Fisher Scientific).

### METHOD DETAILS

#### Mutant library construction

The mutant library was constructed based on the pHW2000 eight-plasmid reverse genetics system for influenza virus.^25^ Mos99 NA-encoding pHW2000 plasmid (pHW2000-Mos99-NA), which was cloned in our previous study,^12^ was used as a PCR template to generate a linearized vector and a library of mutant Mos99 NA inserts. The linearized vector was generated using 5′-CGTACGTCTCAACACACGGAGCGCCCGGGGCCCTCT-3′ and 5′-CGTACGTCTCATAACTGCTAGCGTTAACAGCTTGGG-3′ as primers. Inserts were generated by two batches of PCRs, followed by overlapping PCRs. The first batch of PCRs consisted of 48 reactions, each with an equal molar mix of eight primers as the forward primer and a universal reverse primer 5′-CGTACGTCTCAGTGTGGCTGCGATGGTGGCGTT-3′. The forward primers for the first batch of PCRs are listed in Table S4. These forward primers were named as cassetteX_N, in which X represents the cassette ID and N represents the primer number. Forward primers with the same cassette ID were mixed at equal molar ratio and used in the same PCR. The second batch of PCRs consisted of another 48 reactions, each with a universal forward primer 5′-CGTACGTCTCAGTTAACCGGAGTACTGGTCGAC-3′ and a unique reverse primer as listed in Table S4. Subsequently, 48 overlapping PCRs were performed using the universal forward primer 5′-CGTACGTCTCAGTTAACCGGAGTACTGGTCGAC-3′ and the universal reverse primer 5′-CGTACGTCTCAGTGTGGCTGCGATGGTGGCGTT-3′. For each overlapping PCR, the template was a mixture of 10 ng each of the corresponding products from the first and second batches of PCRs. The complete insert was an equal molar mix of the products of these 48 overlapping PCRs. All PCRs were performed using PrimeSTAR Max polymerase (Takara Bio) according to the manufacturer’s instructions. PCR products were purified using Monarch DNA Gel Extraction Kit (New England Biolabs). Both the vector and the complete insert were digested by BsmBI (New England Biolabs) and ligated using T4 DNA ligase (New England Biolabs). The ligated product was transformed into MegaX DH10B T1R cells (Thermo Fisher Scientific). At least one million colonies were collected. Plasmid mutant libraries were purified from the bacteria colonies using PureLink HiPure Expi Plasmid Purification Kit (Thermo Fisher Scientific). The mutant library contained mutations from residues 82 to 465, which covered 99% of the NA head domain (residues 82 to 469).

#### Rescuing and passaging the mutant library

The virus mutant library was rescued by transfecting a co-culture of HEK 293T and MDCK-SIAT1 cells (ratio of 6:1) at 60% confluence in a T175 flask (175 cm^2^) with six internal segments (PB2, PB1, PA, NP, M, and NS) from H1N1 A/WSN/33, HA segment from H3N2 A/Hong Kong/1/1968, and the Mos99 NA mutant library. Transfection was performed using Lipofectamine 2000 (Thermo Fisher Scientific) according to the manufacturer’s instructions. At 24 h post-transfection, cells were washed twice with PBS and cell culture medium was replaced with OPTI-MEM medium supplemented with 0.8 μg mL^−1^ TPCK-trypsin. The virus mutant library was harvested at 72 h post-transfection, titered by TCID_50_ assay using MDCK-SIAT1 cells, and stored at −80°C until used. To passage the virus mutant library, MDCK-SIAT1 cells in a T75 flask at 80% confluence were washed twice with PBS and infected with the virus mutant library at an MOI of 0.02 in OPTI-MEM medium supplemented with 0.8 μg mL^−1^ TPCK-trypsin. At 2 h post-infection, infected cells were washed twice with PBS and fresh OPTI-MEM medium supplemented with 0.8 μg mL^−1^ TPCK-trypsin was added to the cells. At 24 h post-infection, supernatant was harvested. Each replicate was transfected and passaged independently.

#### Sequencing library preparation

Viral RNA of the post-passaged mutant library was extracted from the supernatant using QIAamp Viral RNA Mini Kit (Qiagen). The extracted RNA was then reverse transcribed to cDNA using Superscript III reverse transcriptase (Thermo Fisher Scientific). The wild-type (WT) plasmid, plasmid mutant library, and the cDNA from the post-infection viral mutant library were amplified by PCR to add part of the adapter sequence required for Illumina sequencing. Six amplicon PCRs were performed for each sample using the following primers:

Amplicon 1: 5′-CACTCTTTCCCTACACGACGCTCTTCCGATCTNNNNNNNAAGGAAATATGCCCCAAACTA-3′ and 5′-GACTGGAGTTCAGACGTGTGCTCTTCCGATCTNNNNNNNCCTATCATGTACTGTGTCATT-3′

Amplicon 2: 5′-CACTCTTTCCCTACACGACGCTCTTCCGATCTNNNNNNNACACTAAACAACGGGCATTCA-3′ and 5′-GACTGGAGTTCAGACGTGTGCTCTTCCGATCTNNNNNNNACCAATACTATCTACAAGCCT-3′

Amplicon 3: 5′-CACTCTTTCCCTACACGACGCTCTTCCGATCTNNNNNNNGCTAGCTTCATTTACAATGGG-3′ and 5′-GACTGGAGTTCAGACGTGTGCTCTTCCGATCTNNNNNNNACAGGAGCATTCCTCGACATG-3′

Amplicon 4: 5′-CACTCTTTCCCTACACGACGCTCTTCCGATCTNNNNNNNCCATTGTCAGGAAGTGCTCAG-3′ and 5′-GACTGGAGTTCAGACGTGTGCTCTTCCGATCTNNNNNNNTTCCTCATTGTTAGGATCCAA-3′

Amplicon 5: 5′-CACTCTTTCCCTACACGACGCTCTTCCGATCTNNNNNNNAGCTCCAGCAGTAGCCATTGC-3′ and 5′-GACTGGAGTTCAGACGTGTGCTCTTCCGATCTNNNNNNNACCAGAATAACCGGACCTATT-3′

Amplicon 6: 5′-CACTCTTTCCCTACACGACGCTCTTCCGATCTNNNNNNNCAAGTCATAGTTGACAGAGGT-3′ and 5′-ACTGGAGTTCAGACGTGTGCTCTTCCGATCTNNNNNNNGAACAAATTATATAGGCATGAG-3′

A total of 12 N were included in each amplicon product as unique molecular identifiers (UMIs) to distinguish true mutations from sequencing errors.^22,55,56^ For each sample, the six amplicon PCR products were mixed at equal molar ratio. Subsequently, 3.2 million copies of mixed amplicon PCR products were used as template for a second PCR to add the rest of the adapter sequence and index to the amplicon using primers: 5′-AAT GAT ACG GCG ACC ACC GAG ATC TAC ACX XXX XXX XAC ACT CTT TCC CTA CAC GAC GCT-3′, and 5′-CAA GCA GAA GAC GGC ATA CGA GAT XXX XXX XXG TGA CTG GAG TTC AGA CGT GTG CT-3′. Positions annotated by an “X” represented the nucleotides for the index sequence. All PCRs were performed using using KOD Hot Start DNA polymerase (MilliporeSigma) according to the manufacturer’s instructions. PCR products were purified using PureLink PCR Purification Kit (Thermo Fisher Scientific). The final PCR products were submitted for the next-generation sequencing using one lane of NovaSeq SP PE150.

#### Sequencing data analysis

Sequencing data was obtained in FASTQ format and analyzed using a custom snakemake pipeline.^57^ Firstly, paired-end reads with the same UMI were grouped together. Groups with only one read were discarded. Groups with reads that had different lengths were also discarded. A consensus read was generated for each group of reads with an 80% minimum frequency cutoff. In other words, for each position, the same nucleotide needed to be shared among at least 80% of the reads, otherwise the group would be discarded. Subsequently, primer sequences were trimmed using cutadapt^58^ and each consensus read was assigned to one of the six amplicons based on the primer sequence. Consensus reads that were not assigned to any amplicon were discarded. Forward and reverse consensus reads were then merged by FLASH^59^ using parameters: −m 30 −M 70 −I. Merged consensus reads were parsed by SeqIO module in BioPython^60^ and translated into amino acid sequences. A merged consensus reads were removed if its sequence length was different from the referenced amplicon. Afterward, mutations were called by comparing the amino acid sequences of each merged consensus read to that of the referenced amplicon. Merged consensus reads that contained more than one amino acid mutations would be discarded. The frequency of each mutation in individual samples was computed. The frequency of mutation *i* in sample s was calculated as follows:

(1) $${\mathrm{frequency}}_{i,s}=\frac{{\mathrm{read count}}_{i,s}+1}{\underset{k\in s}{\sum }{\mathrm{read count}}_{k,s}+1}$$

A mutation would be discarded if its frequency in the plasmid mutant library was not at least 6-fold higher than its frequency in the WT sample. This filter aimed to prevent error hotspots in reverse transcription, PCR, and next-generation sequencing from confounding our fitness calculation. In addition, a mutation would be discarded if its read count in the plasmid mutant library was 10 or less. After these filter steps, fitness calculation was performed.

For each replicate, the enrichment and fitness of a mutation *i* in amplicon *amp* was calculated as follows:

(2) $${\mathrm{enrichment}}_{i}=\frac{{\mathrm{read count}}_{input,i}+1}{{\mathrm{read count}}_{post-passaged,i}+1}$$

(3) $${\mathrm{fitness}}_{i}=log_{10}\left({\mathrm{enrichment}}_{i}\right)-\frac{1}{N_{silent,amp}}\underset{k\in silent,amp}{\sum }log_{10}\left({\mathrm{enrichment}}_{k}\right)$$

where the *read count*_*input,i*_ represents the read count of mutation *i* in the plasmid mutant library, and *read count*_*post–passaged,i*_ represents the read count of mutation *i* in the post-passaged mutant library. A pseudocount of 1 was added to the read counts to avoid division by zero. The fitness for mutation *i* was in log_10_ scale and normalized to the mean fitness of silent mutations in amplicon *amp*, such that the mean fitness of silent mutations was 0. The final fitness was computed as the averaged fitness from the two biological replicates.

#### Protein expression and purification

The NA head domains, which contained residues 82 to 469, were fused to an N-terminal gp67 signal peptide, 6 × His-tag, a vasodilator-stimulated phosphoprotein (VASP) tetramerization domain, and a thrombin cleavage site. Recombinant bacmid DNA that carried Mos99 NA ectodomain was generated using the Bac-to-Bac system (Thermo Fisher Scientific) according to the manufacturer’s instructions. Baculovirus was generated by transfecting the purified bacmid DNA into adherent Sf9 cells using Cellfectin reagent (Thermo Fisher Scientific) according to the manufacturer’s instructions. The baculovirus was further amplified by passaging in adherent Sf9 cells at a multiplicity of infection (MOI) of 1. Recombinant NA head domains were expressed by infecting 1 L of suspension Sf9 cells at an MOI of 1. On day 3 post-infection, Sf9 cells were pelleted by centrifugation at 4,000 × g for 25 min, and soluble recombinant NA was purified from the supernatant by affinity chromatography using Ni Sepharose excel resin (Cytiva) and then size exclusion chromatography using a HiLoad 16/100 Superdex 200 prep grade column (Cytiva) in 20 mM Tris-HCl pH 8.0, 100 mM NaCl, and 10 mM CaCl_2_. For crystallography, recombinant NA was further digested by thrombin (MilliporeSigma) for three weeks in 4°C using 15 U thrombin per mg of recombinant NA. The thrombin-digested recombinant NA was incubated with TALON metal affinity resin (Takara) for 2 h. The thrombin-digested recombinant NA in the flow-through and 10 mM imidazole wash was purified by size exclusion chromatography using a HiLoad 16/100 Superdex 200 prep grade column (Cytiva) in 20 mM Tris pH 8.0, 100 mM NaCl, and 10 mM CaCl_2_.

#### Crystallization and structural determination

Crystallization screening was performed using the JCSG Core Suites I-IV (Rigaku) with thrombin-digested NA at 7 μg mL^−1^. Sitting drop for crystallization screening was set up by equal volume of precipitant and protein solution using the Crystal Gryphon (Art Robbins Instruments). Crystallization screens were incubated at 18°C. Initial hits were further optimized using the sitting drop method at 18°C, with 350 μL reservoir solution and 1:1 ratio of precipitant and protein solution. The crystallization condition was optimized to 1.9 M NH_4_SO_4_, 0.2 M Li_2_SO_4_, 0.1 M Tris pH 7.0. Large crystals grew over 3 days and were subsequently soaked for 3 h at 18°C in the precipitant solution supplemented with 20 mM N-acetylneuraminic acid. Crystals were soaked in precipitant solution supplemented with 20% glycerol as cryoprotectant prior to vitrification in liquid nitrogen.

Data were collected at the Advanced Photon Source (APS) at Argonne National Laboratory via the Life Science Collaborative Access Team (LS-CAT) at beamline 21-ID-D. Initial diffraction data were indexed, integrated, and scaled using autoPROC.^50^ The structure was solved by molecular replacement using Phaser-MR included in the Phenix suite,^51^ with the apo Mos99 NA structure (PDB: 7U4F)^13^ as the replacement model. The structure was further refined using REFMAC5^52^ and was manually built in COOT.^53^ Ramachandran statistics were calculated using MolProbity.^54^

#### Residue classification

DSSP^61^ was used to compute the solvent exposure surface area (SASA) for individual residues in the Mos99 NA head domain (PDB: 7U4F).^13^ Relative solvent accessibility (RSA) was computed by dividing the SASA by the theoretical maximum allowed solvent accessibility of the corresponding amino acid.^62^ SASA and RSA were computed using both monomeric and tetrameric forms of Mos99 NA head domain. Active site was defined as previously described,^6^ which included residues 118, 151, 152, 224, 276, 292, 371, and 406. Similarly, antigenic regions were defined as previously described,^10^ which included residues 153, 197, 198, 199, 328, 329, 330, 331, 332, 333, 334, 335, 336, 339, 340, 341, 342, 343, 344, 345, 346, 347, 367, 368, 369, 370, 400, 401, 402, 403, 431, 432, 433, and 434. The remaining residues were classified into “buried”, “interface”, and “exposed”:

Buried residues: RSA_monomer_ < 0.2.
Interface residues: RSA_monomer_ ≥ 0.2 and (RSA_monomer_ – RSA_tetramer_)/RSA_monomer_ ≤ 0.5.
Exposed: RSA^monomer^ ≥ 0.2 and (RSA_monomer_ – RSA_tetramer_)/RSA_monomer_ ≤ 0.5.

#### Sequence logo analysis

Sequence logos were generated by Logomaker in Python.^63^ All sequences were downloaded from Global Initiative for Sharing Avian Influenza Data (GISAID).^64^ Sequences with ambiguous amino acids were discarded. The representative strains for different NA subtypes were (GISAID accession numbers in parentheses):

N1: H1N1 A/California/07/2009 (EPI185379)
N2: H3N2 A/Moscow/10/1999 (EPI362897)
N3: H2N3 A/swine/Missouri/2124514/2006 (EPI126570)
N4: H10N4 A/mink/Sweden/E12665/84 (EPI16618)
N5: H12N5 A/mallard-duck/ALB/60/1976 (EPI86400)
N6: H3N6 A/chicken/Nanchang/7-010/2000 (EPI89011)
N7: H10N7 A/mallard/ALB/196/1996 (EPI88485)
N8: H3N8 A/duck/Ukraine/1/1963 (EPI3107)
N9: H11N9 A/tern/Australia/G70C/1975 (EPI129556)

#### FoldX

We applied the FoldX force field^34^ to predict the change of thermostability (ΔΔG) of NA protein upon single amino acid substitutions. The crystal structure of Mos99 NA in complex with sialic acid that was determined in this study was used as the input for FoldX. Atoms of sialic acid were parameterized using pyFoldX^35^ with default parameters. We first prepared the complex structure by optimizing atomic details using FoldX RepairPDB. Next, we substituted each amino acid of the WT sequence to the other 19 amino acids with the FoldX BuildModel based on the optimized structure and output energy values before and after mutation. ΔΔG was computed by the difference of energy values between the WT and the mutant. The above procedure was repeated 10 times to obtain an average ΔΔG estimation. While our crystal structure represented the native tetrameric form of the Mos99 NA, we also performed the same ΔΔG prediction procedure for the monomeric form by using one protomer of the crystal structure as the input.

#### MSA transformer

MSA Transformer is a transformer protein language model pre-trained with the mask language modeling objective.^40^ MSA Transformer takes a set of aligned sequences as input, and outputs the probability that an amino acid occurs at the masked position in a protein given the surrounding sequence context. It was trained on a dataset that contained 26 million multiple sequence alignments, each of which was generated by searching an Uniref. 50 sequence against the UniClust30 database with HHblits. The model was trained on 32 V100 GPUs for 450,000 updates. To infer the mutational fitness of Mos99 NA, 66,562 full-length human H3N2 NA sequences from 1968 to 2020 from were downloaded from the GISAID database and used as the input MSA. Next, we masked individual positions in the Mos99 NA sequence one at a time and inferred the fitness for each single amino acid mutation at the given position by comparing its predicted probability to that of the WT amino acid^65^:

$$\mathrm{Inferred fitness}=\log\;p\left(x_{i}=x_{i}^{mut}\right)-\log\;p\left(x_{i}=x_{i}^{WT}\right)$$

### QUANTIFICATION AND STATISTICAL ANALYSIS

The p values reported in Figure 2B and all correlation coefficients in this study were computed by the paired Student’s t-test using the R software package.

## Supplementary Material

1

2

3

4

## Figures and Tables

**Table:** KEY RESOURCES TABLE

REAGENT or RESOURCE	SOURCE	IDENTIFIER
Antibodies		
6x-His Tag Monoclonal Antibody (HIS.H8)	Thermo Fisher Scientific	Cat#14-6657-82; RRID: AB_2572898
HRP Rat Anti-Mouse Ig, κ Light Chain	BD Biosciences	Cat#559751; RRID: AB_397315
Chemicals, peptides, and recombinant proteins		
Sodium chloride (NaCl)	Fisher Scientific	Cat#S271-500
Hydrochloric Acid, ACS, 12 M	Fisher Scientific	Cat#S25358
Tris Base	Fisher Scientific	Cat#BP152-500
Imidazole	Fisher Scientific	Cat#A10221
Calcium Chloride, Dihydrate	Millipore	Cat#208291-250GM
DpnI	New England Biolabs	Cat#R0176L
BsmBI-v2	New England Biolabs	Cat#R0739L
T4 DNA Ligase	New England Biolabs	Cat#M0202L
Lipofectamine 2000	Thermo Fisher Scientific	Cat#11668-019
Cellfectin II Reagent	Gibco	Cat#10362-100
TPCK-Trypsin	Thermo Fisher Scientific	Cat#20233
RNaseOUT	Thermo Fisher Scientific	Cat#10777019
6′-a-Sialyl-N-acetyllactosamine sodium salt	Biosynth	Cat#OS09313
JCSG Core Suite 1 Screen	Rigaku	Cat#1009842
JCSG Core Suite 2 Screen	Rigaku	Cat#1009843
JCSG Core Suite 3 Screen	Rigaku	Cat#1009844
JCSG Core Suite 4 Screen	Rigaku	Cat#1009845
Sf-900 II SFM	Thermo Fisher Scientific	Cat#10902088
DMEM medium	Thermo Fisher Scientific	Cat#11995065
Opti-MEM I Reduced Serum Medium	Thermo Fisher Scientific	Cat#31985070
GlutaMAX Supplement	Thermo Fisher Scientific	Cat#35050061
NEAA mixture (100x)	Lonza	Cat#13-114E
Trypsin-EDTA (0.25%), phenol red	Thermo Fisher Scientific	Cat#25200056
Penicillin-Streptomycin	Thermo Fisher Scientific	Cat#15140122
Fetal Bovine Serum (FBS)	Thermo Fisher Scientific	Cat#16000044
Phosphate-buffered saline (PBS), 1X	VWR	Cat#21-040-CM
NEB 5-alpha Competent *E. coli*	New England Biolabs	Cat#C2987H
MAX Efficiency DH10Bac Competent Cells	Thermo Fisher Scientific	Cat#10361012
MegaX DH10B T1R Electrocomp Cells	Thermo Fisher Scientific	Cat#C640003
NA protein sequences	GISAID; http://gisaid.org/	N/A
Ni Sepharose excel resin	Cytiva	Cat#17371202
XK 16/100 Superdex 200 pg	Cytiva	Cat#90100137
Critical commercial assays		
PrimeSTAR Max DNA Polymerase	Takara	Cat#R045A
KOD Hot Start DNA Polymerase	EMD Millipore	Cat#71086-3
PureLink PCR Purification Kit	Thermo Fisher Scientific	Cat#K310002
QIAprep Spin Miniprep Kit	Qiagen	Cat#27106
PureLink HiPure Plasmid Miniprep Kit	Thermo Fisher Scientific	Cat#K210003
Monarch DNA Gel Extraction Kit	New England Biolabs	Cat#T1020L
Superscript III reverse transcriptase	Thermo Fisher Scientific	Cat#18080044
Deposited data		
Raw sequencing reads	This study	BioProject: PRJNA857746
X-ray coordinates and structure factors	This study	PDB: 8DWB
Experimental models: Cell lines		
Sf9 cells	ATCC	ATCC CRL-1711
MDCK-SIAT1 cells	Sigma-Aldrich	Cat#05071502-1VL
HEK 293T cells	N/A	N/A
Recombinant DNA		
pHW2000-chimeric HK68 HA	This study	N/A
pHW2000-Mos99 NA	This study	N/A
WSN 8-plasmid reverse genetics	Neumann et al.^25^	N/A
pFastBac-Mos99 NA	Lei et al.^13^	N/A
Software and algorithms		
Octet analysis software 9.0	Fortebio	N/A
Python	https://www.python.org/	N/A
R	https://www.r-project.org/	N/A
autoPROC	Vonrhein et al.^50^	N/A
Phenix suite	Adams et al.^51^	N/A
REFMAC5	Murshudov et al.^52^	N/A
COOT	Emsley et al.^53^	N/A
MolProbity	Chen et al.^54^	N/A
FoldX	Delgado et al.^34^	N/A
pyFoldX	Radusky and Serrano^35^	N/A
MSA Transformer	Rao et al.^40^	N/A
Custom scripts	This study	https://doi.org/10.5281/zenodo.7416749

## Highlights

- Deep mutational scanning (DMS) of human influenza H3N2 neuraminidase head domain
- DMS result correlates with mutational fitness inferred from natural sequences
- Mutational fitness effects correlate with protein stability effects (ΔΔG)
