# Mutational fitness landscape of human influenza H3N2 neuraminidase

**DOI:** 10.1016/j.celrep.2023.113356

**Published:** 2023-10-17

**Authors:** Ruipeng Lei, Andrea Hernandez Garcia, Timothy J.C. Tan, Qi Wen Teo, Yiquan Wang, Xiwen Zhang, Shitong Luo, Satish K. Nair, Jian Peng, Nicholas C. Wu

In the originally published version of this article, the numerator and the denominator of Equation 2 were mistakenly swapped. The article has been updated to reflect the correct numerator and denominator, and the conclusions of the paper were unaffected by the initial error. The authors sincerely regret the error.

