# Supplementary material for: Mutational fitness landscape of human influenza H3N2 neuraminidase
Source: Cell Rep. Author manuscript; Available in PMC 2023 Feb 16. (PMC9931530; doi:10.1016/j.celrep.2022.111951)
Supplement: 1 [file NIHMS1870513-supplement-1.pdf]

Cell Reports, Volume *42*

Supplemental information

Mutational fitness landscape

# of human influenza H3N2 neuraminidase

Ruipeng Lei, Andrea Hernandez Garcia, Timothy J.C. Tan, Qi Wen Teo, Yiquan Wang, Xiwen Zhang, Shitong Luo, Satish K. Nair, Jian Peng, and Nicholas C. Wu

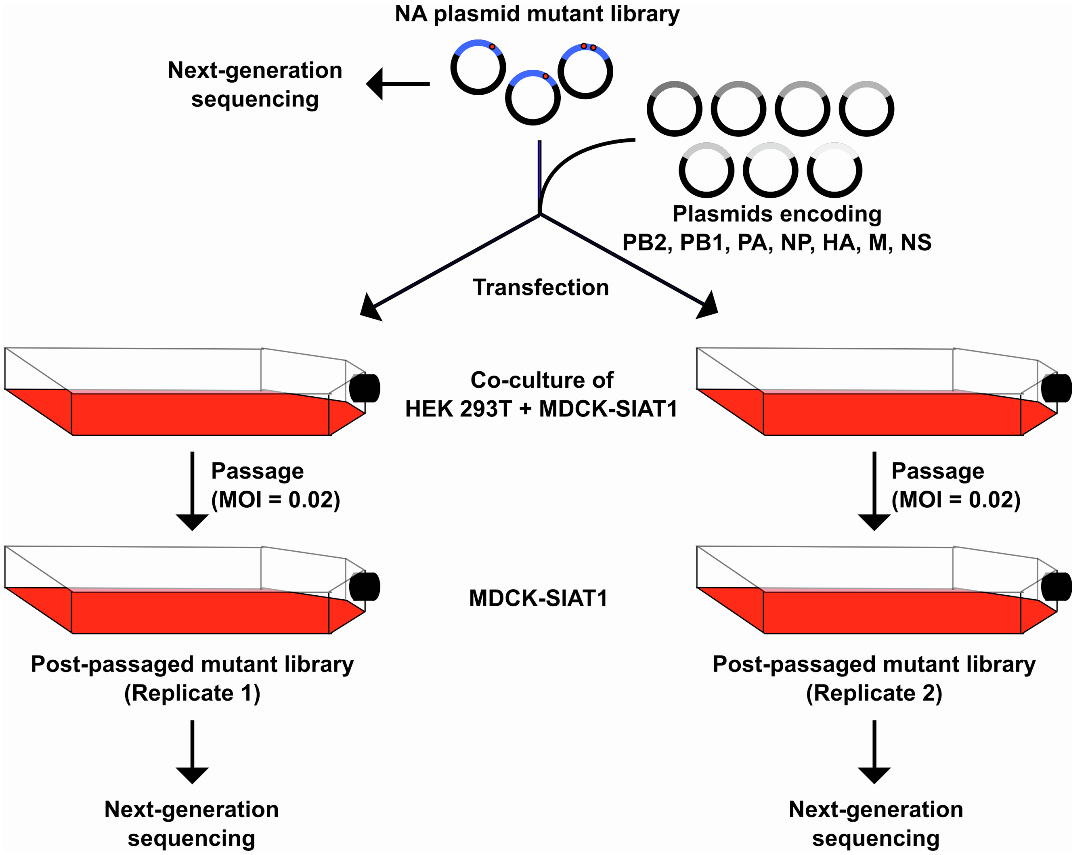

Figure S1. Experimental design, Related to Figure 1. The Mos99 NA virus mutant library was rescued by transfecting the Mos99 NA plasmid mutant library along with plasmids encoding six internal segments from A/WSN/33 (H1N1) and HA segment from A/Hong Kong/1/1968 (H3N2) into a co-culture of HEK 293T cells and MDCK-SIAT1 cells. The Mos99 NA virus mutant library was then passaged once in MDCK-SIAT1 cells at a multiplicity of infection (MOI) of 0.02 to generate the post-passaged mutant library. A biological replicate of transfection and passaging was performed using the same NA plasmid mutant library. The NA plasmid mutant library as well as both replicates of the post-passaged mutant library were analyzed by next-generation sequencing.

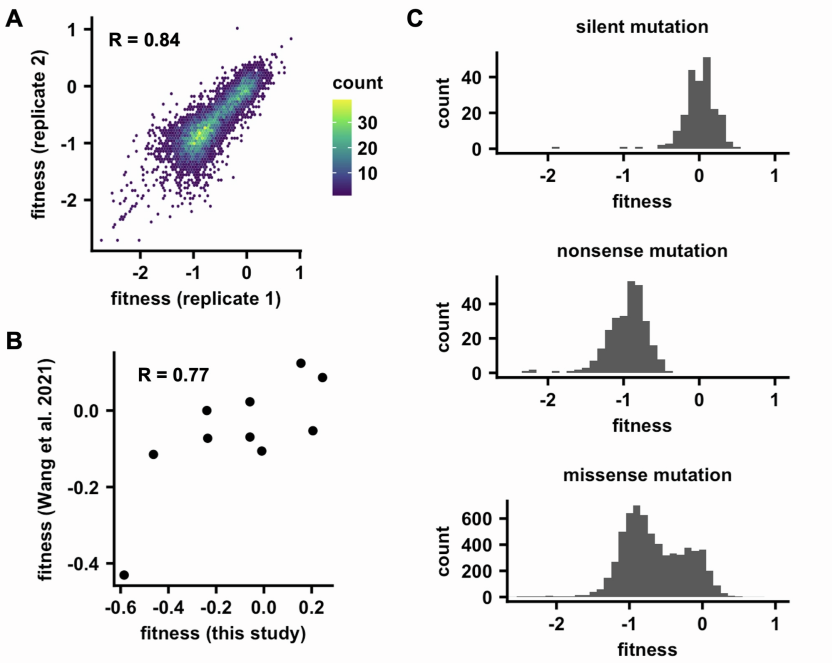

Figure S2. Reproducibility and quality of the deep mutational scanning data, Related to Figure 1. (A) Correlation of fitness measurements for individual mutations between two biological replicates is shown as a density scatterplot. **(B)** Correlation of fitness measurements between our deep mutational scanning result and our previous study that focused on a seven-residue antigenic site[21] is shown as a scatterplot. Each data point represents one of the 10 single mutations that have fitness effect measured in both this study and our previous study[21]. (**A-B**) The Pearson correlation coefficient (R) is indicated. **(C)** The distributions of fitness effects for missense, nonsense, and silent mutations are shown as histograms.

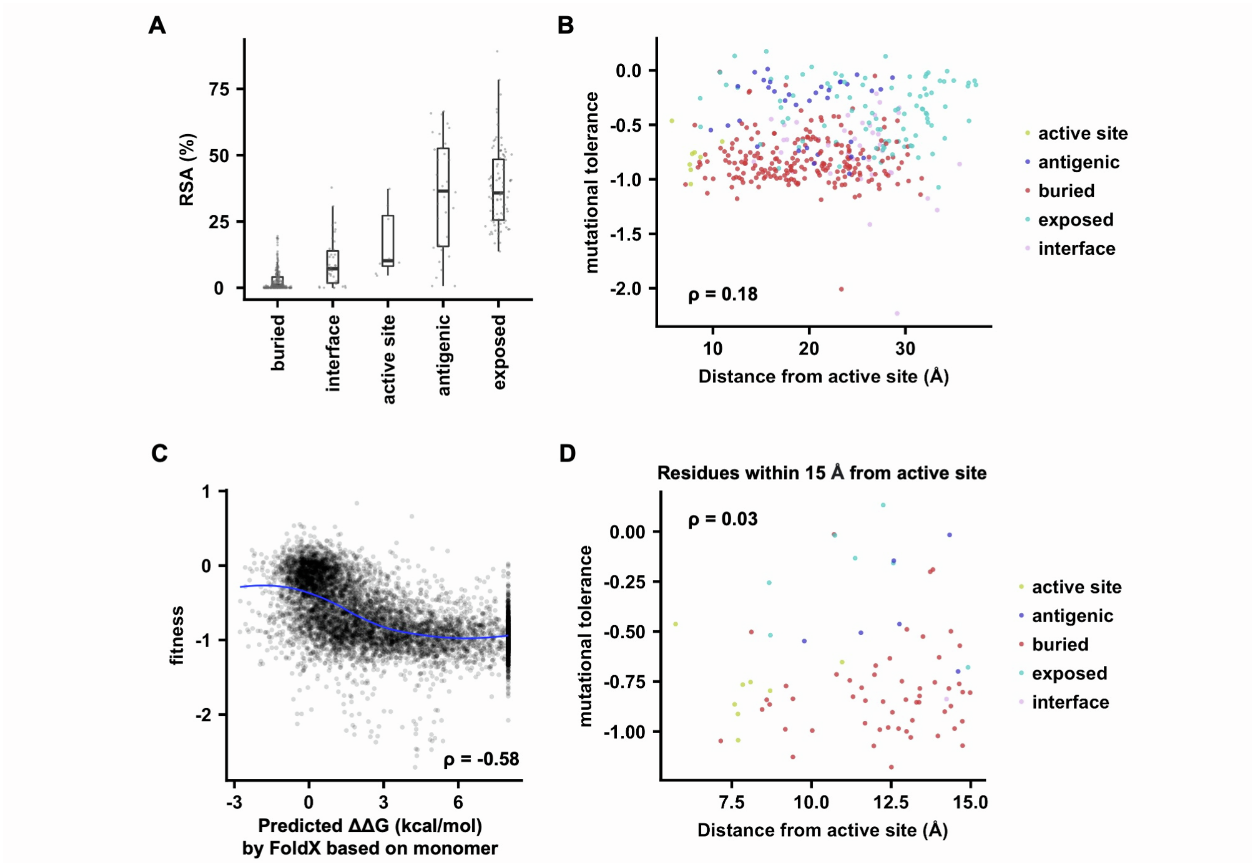

Figure S3. Mutational tolerance correlates weakly with the distance from active site, Related Figure 2. (A) The distribution of mutational tolerance for each residue category is shown as a box plot. Each data point represents the mutational tolerance of one residue. **(B)** The relationship between mutational tolerance and the distance from active site is shown. Each data point represents one residue and colored according to the residue type. **(C)** Same as Figure 4A, except monomer is used instead of tetramer for computing the predicted stability effect (ΔΔG). Mutations with predicted ΔΔG > 8 kcal mol^{-1} are shown as 8 kcal mol^{-1}. **(D)** Same as panel A, except only residues within 15 Å from the active are included. The Spearman's rank correlation coefficient (ρ) is indicated.

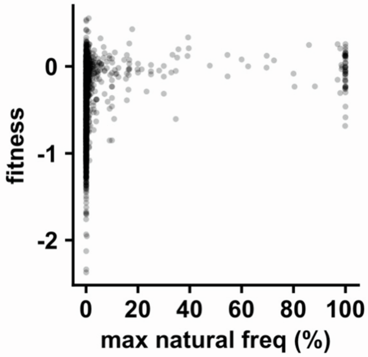

Figure S4. Fitness of naturally occurring mutations, Related to Figure 4. For each mutation, its natural occurrence frequency in each year from 1968 to 2020 was computed. Maximum natural occurrence frequency represents the highest annual frequency that a given mutation has reached. The relationship between the maximum natural occurrence frequency and fitness is shown. Each data point represents one mutation.

Table S2. X-ray data collection and refinement statistics, Related to Figures 2-4.

	Mos99 NA complex with N-Acetylneuraminic acid
**Data collection**	
Wavelength (Å)	1.12723
Resolution (Å)	1.602
Resolution Range [a]	100.889-1.602 (1.607-1.602)
Space group	I 4 2 2
Cell dimensions	
a, *b*, *c* (Å)	136.22, 136.22, 150.15
α, *β*, *γ* (°)	90.00, 90.00, 90.00
Total reflections	1,475,553
Unique reflections	92,200
Multiplicity [a]	16.0 (13.1)
Completeness (%) [a]	100.0 (100.0)
$\langle I/\sigma_I \rangle$ [a]	14.6 (2.0)
R_{merge} (%) [a]	12.3 (106.5)
R_{meas} (%) [a]	12.7 (110.9)
$CC_{1/2}$ [a]	0.999 (0.913)
**Refinement**	
Resolution (Å)	35.00-1.60
No. reflections	87,607
R_{work}^{c} / R_{free}^{d}	0.169/0.182
No. atoms	3,428
Protein	3,055
Water	236
Sugars/Ligand	137
B-factors	
Protein	16.8
Sugars/Inhibitor	25.3
Water	22.4
Bond lengths (Å)	0.014
Bond angles (°)	1.93
Favored	95.85
Outliers	0.0
**PDB code**	8DWB

[a] Numbers in parentheses refer to the highest resolution shell.
[b] $R_{merge} = \Sigma |I_i - \langle I_i \rangle| / \Sigma I_i$ where I_i = the intensity of the *i*th reflection and $\langle I_i \rangle$ = mean intensity.
[c] $R_{work} = \Sigma |F_o - F_c| / \Sigma |F_o|$, where F_o and F_c are the observed and calculated structure factors, respectively.
[d] R_{free} was calculated as for R_{work}, but on a test set comprising 5% of the data excluded from refinement.
